# Causal relationship between immune cells and prostate cancer: a Mendelian randomization study

**DOI:** 10.3389/fcell.2024.1381920

**Published:** 2024-03-19

**Authors:** Zhipeng Ye, Xinpei Deng, Jinhui Zhang, Ruonan Shao, Cailu Song, Jianfu Zhao, Hailin Tang

**Affiliations:** ^1^ State Key Laboratory of Oncology in South China, Guangdong Provincial Clinical Research Center for Cancer, Sun Yat-Sen University Cancer Center, Guangzhou, China; ^2^ Department of Oncology, The First Affiliated Hospital of Jinan University, Guangzhou, China

**Keywords:** prostate cancer, immune cells, Mendelian randomization, single nucleotide polymorphism, genome-wide association studies

## Abstract

**Introduction:**

Despite the abundance of research indicating the participation of immune cells in prostate cancer development, establishing a definitive cause-and-effect relationship has proven to be a difficult undertaking.

**Methods:**

This study employs Mendelian randomization (MR), leveraging genetic variables related to immune cells from publicly available genome-wide association studies (GWAS), to investigate this association. The primary analytical method used in this study is inverse variance weighting (IVW) analysis. Comprehensive sensitivity analyses were conducted to assess the heterogeneity and horizontal pleiotropy of the results.

**Results:**

The study identifies four immune cell traits as causally contributing to prostate cancer risk, including CD127- CD8+ T cell %CD8+ T cell (OR = 1.0042, 95%CI:1.0011–1.0073, *p* = 0.0077), CD45RA on CD39+ resting CD4 regulatory T cell (OR = 1.0029, 95%CI:1.0008–1.0050, *p* = 0.0065), CD62L− Dendritic Cell Absolute Count (OR = 1.0016; 95%CI:1.0005–1.0026; *p* = 0.0039), CX3CR1 on CD14+ CD16− monocyte (OR = 1.0024, 95%CI:1.0007–1.0040, *p* = 0.0060). Additionally, two immune cell traits are identified as causally protective factors: CD4 on monocyte (OR = 0.9975, 95%CI:0.9958–0.9992, *p* = 0.0047), FSC-A on plasmacytoid Dendritic Cell (OR = 0.9983, 95%CI:0.9970–0.9995, *p* = 0.0070). Sensitivity analyses indicated no horizontal pleiotropy.

**Discussion:**

Our MR study provide evidence for a causal relationship between immune cells and prostate cancer, holding implications for clinical diagnosis and treatment.

## Introduction

Prostate cancer is a prevalent malignant tumor in elderly men, ranking as the most common solid malignancy in men in western countries, with an increasing incidence year by year (Rebello et al., 2021). Current research indicates that the occurrence of prostate cancer is primarily associated with factors such as age, hormones, race, and genetics (Bergengren et al., 2023). However, its etiology and pathogenesis are not fully understood. Treatment for prostate cancer primarily includes surgery, radiation therapy, and androgen deprivation therapy (Cha et al., 2020). Emerging treatment modalities have provided patients with a greater range of options. For example, the use of nanomaterials in conjunction with circRNA enhance the sensitivity of tumor cells to treatment (Ghorbani et al., 2023; Su et al., 2023; Wang et al., 2023; Xie et al., 2023; Zetrini et al., 2023; Zhou et al., 2023). However, currently the efficacy for recurrent, drug-resistant, and metastatic prostate cancer is limited (Antonarakis et al., 2010; Gao et al., 2023; Guo et al., 2023; Sooi et al., 2023; Su et al., 2023). Therefore, it is imperative to investigate the etiology, pathogenesis, and explore new treatment methods for prostate cancer.

Increasing research indicates that immune cells were involved in the development of prostate cancer. Various cell types involved in the regulation of prostate cancer have been identified (Fridlender et al., 2009; Sagnak et al., 2011; Shi et al., 2023). NK cells and CD8^+^ T lymphocytes are pivotal forces in anti-tumor immunity, effectively eliminating cancer cells. Conversely, tumor-associated macrophages and other cells exert inhibitory effects on anti-tumor immunity, and their excessive activation may be associated with the occurrence and progression of tumors (Luo et al., 2021; Shi et al., 2023). While there is a preliminary understanding of the roles of certain immune cell types in the pathogenesis of prostate cancer, the specific functions of various subtypes of immune cells and whether there is a causal relationship between these cells and tumor development remain unclear. Clarifying the causal relationship between immune cells and the onset of prostate cancer is a critical topic in current prostate cancer research.

However, the majority of research methods currently employed still face significant limitations in establishing a causal relationship between these factors. Mendelian randomization (MR), utilizing genetic variations as instrumental variables, is a valuable tool for establishing causal relationships. MR improves study validity by reducing bias and enabling causal inference in experimental designs. Mendelian randomization analysis offers advantages over randomized controlled trials (RCTs) by utilizing genetic variants as instrumental variables, providing insights into long-term exposures and outcomes, reducing confounding bias inherent in observational studies, and offering cost-effective alternatives in situations where RCTs are impractical or unethical (Smith and Ebrahim, 2003; Larsson and Burgess, 2022). In this study, we employ MR to investigate the causal relationship between immune cells and prostate cancer. This approach is advantageous for illustrating the relationship between immune cells and prostate cancer, laying the groundwork for immunotherapeutic interventions in prostate cancer.

## Methods

### Data sources

This study utilized a population-based immune profiling analysis reported in the Nature Genetics journal. The research included a cohort of 3,757 individuals from the Sardinian population. The comprehensive investigation encompassed a wide range of 731 immunophenotypes, comprising absolute cell counts (n = 118), median fluorescence intensities (n = 389), morphological parameters (n = 32), and relative cell counts (n = 192) (Orru et al., 2020).

**FIGURE 1:**
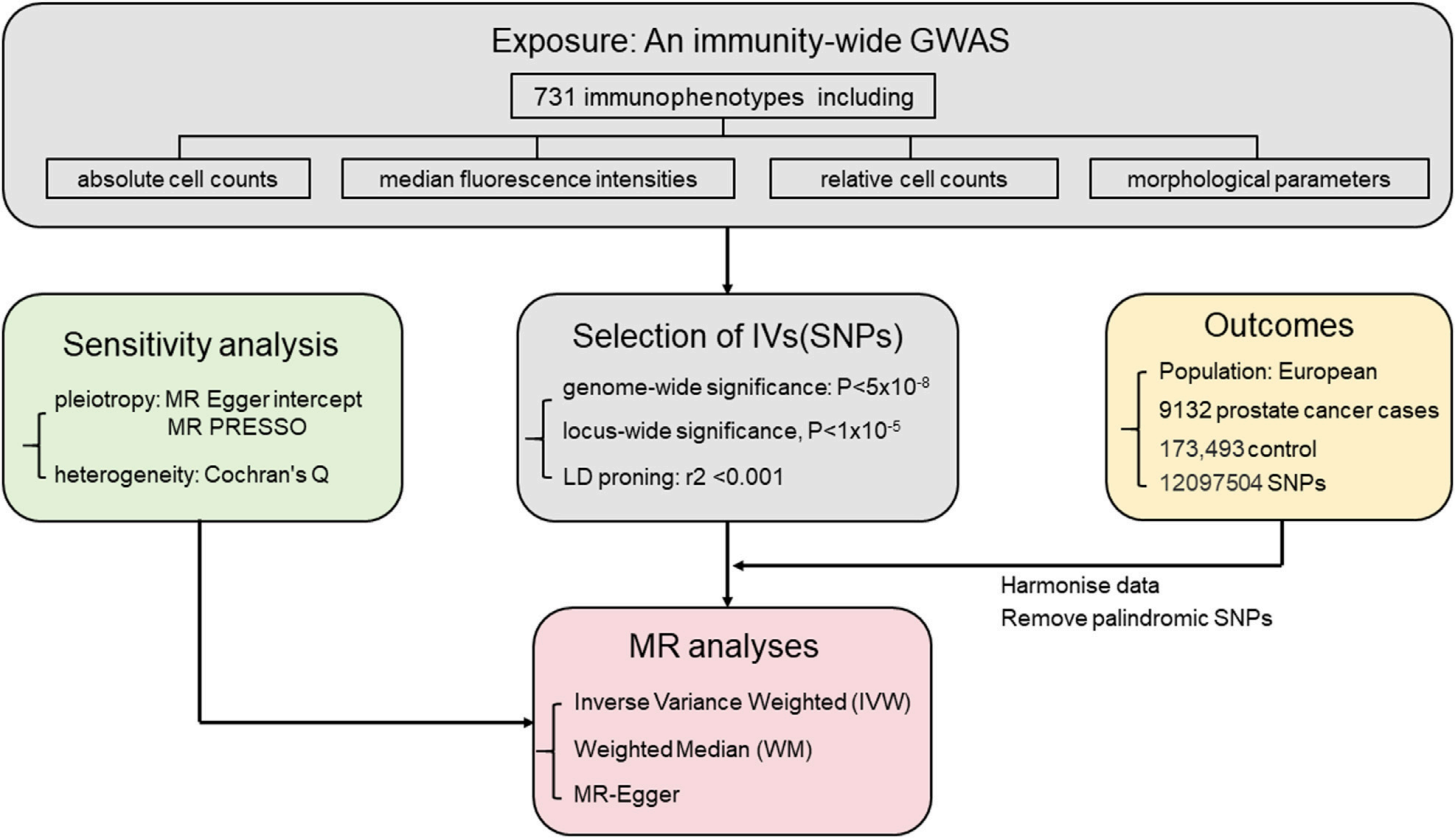
Mendelian randomization study workflow on the association between immune cell types and prostate cancer.

The prostate cancer data used in this study were sourced from the Integrative Epidemiology Unit Open GWAS database (https://gwas.mrcieu.ac.uk/). The study included 9,132 European male prostate cancer patients as the study group and 173,493 European males without prostate cancer as the control group. A total of 12,097,504 SNPs were screened for their impact on prostate cancer. The diagnostic criteria for prostate cancer are derived from ICD-10 code C61 and ICD-9 code 185 (Kimberley Burrows, 2021). Figure 1 illustrates the study’s specific research approach, while Table 1 provides specific details on data sources and features.

**TABLE 1:** Detailed information on the analyzed data.

Exposure or outcome	Sample size	Population	Data source	PMID
Immune cell types	3,757	European	https://pubmed.ncbi.nlm.nih.gov/32929287/	32929287
prostate cancer	9,132	European	https://gwas.mrcieu.ac.uk/datasets/ieu-b-4809/	—

### Instrumental variables (IVs)

**FIGURE 2:**
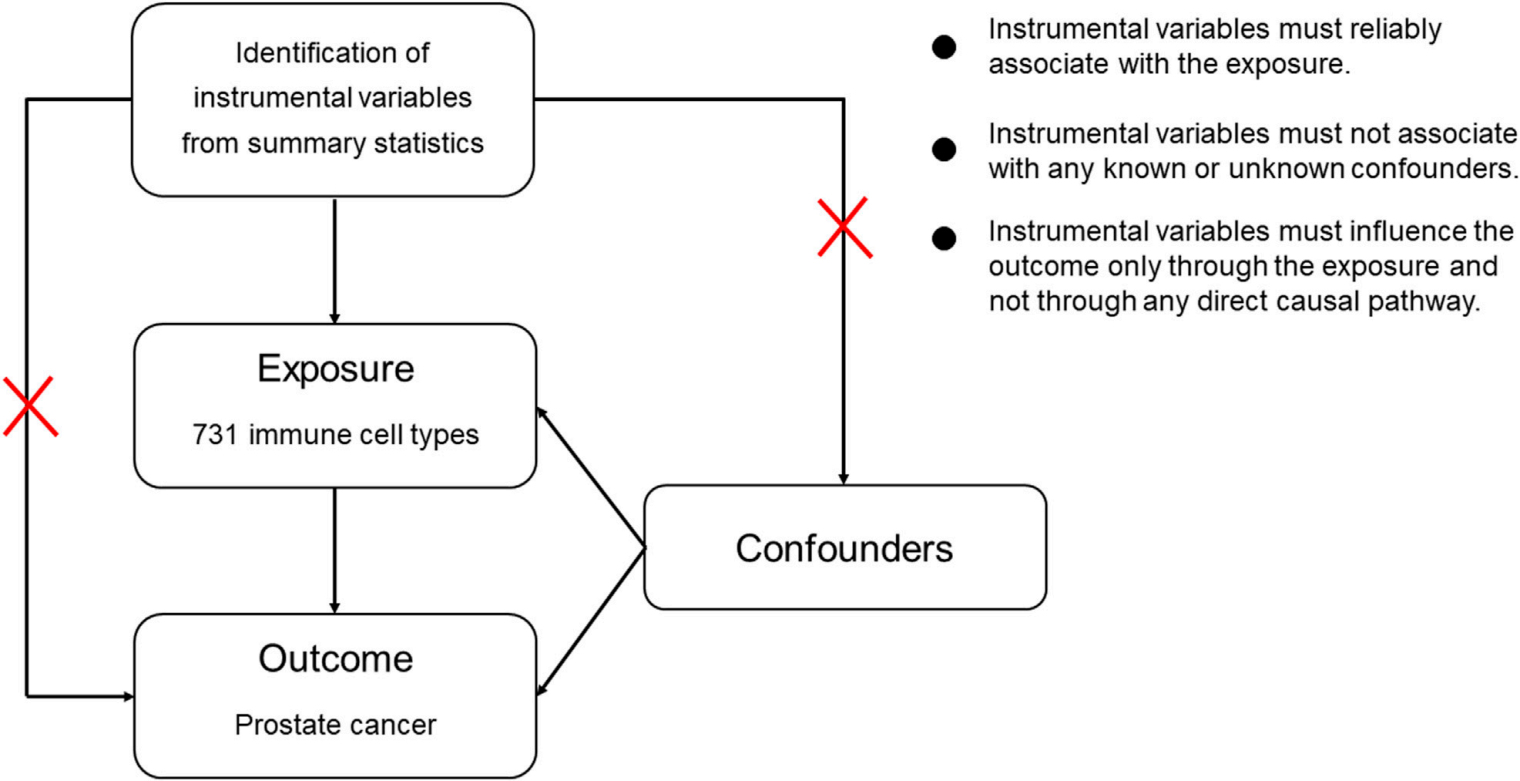
Assumptions in MR studies: a brief overview.

MR employs genetic variants as instrumental variables. The selection of instrumental variables needs to satisfy three assumptions: 1) the genetic variants have strong association with the exposure factor; 2) the genetic variants were independent from confounding factors. 3) the genetic variants affect the outcome through the exposure factor (Smith and Ebrahim, 2004; Richmond and Davey Smith, 2022). The process of selecting genetic variants as instrumental variables is illustrated in Figure 2.

The selection and analysis of IVs for immune traits were conducted meticulously, employing a significance level of 1 × 10^−5^. To ensure the independence of loci, a clumping window of 10,000 kb and a linkage disequilibrium (LD) threshold of r2 < 0.001 were employed. The implementation was carried out using the “TwoSampleMR” package and 1,000 Genomes EUR data. Palindrome SNPs may introduce uncertainty regarding the effect allele in GWAS. To ensure reliability, we excluded palindrome SNPs with effect allele frequencies between 0.3 and 0.7. Additionally, instrument strength was assessed using F-statistics, where a variance ratio (b^2^/se^2^) exceeding 10 indicates minimal weak instrument bias (Burgess et al., 2011).

### Statistical analysis

In this research, various analytical methods including the Inverse Variance Weighted (IVW) method, weighted median method, and MR-Egger regression analysis were employed. The IVW method, widely used in MR studies for its excellent accuracy in effect estimation, was applied in this study with a higher screening threshold (*p*-value <0.01) to ensure result accuracy. Random-effects IVW provides an unbiased estimate by considering heterogeneity among studies and appropriately weighting the effects based on their precision. The IVW method was chosen as the primary research method used in this study (Hemani et al., 2018b). The MR Egger method accurately evaluates causal relationships, effectively addresses sample selection bias, and enhances statistical power and robustness of data (Verbanck et al., 2018). The weighted median approach provides a reliable estimate by accounting for the distribution of weights assigned to each data point (Hemani et al., 2018b). Cochran’s Q test was used to assess heterogeneity (Bowden et al., 2019). MR Egger intercept analysis serves to assess and correct for potential bias caused by horizontal pleiotropy. MR-PRESSO analysis method is designed to detect and correct for horizontal pleiotropy (Verbanck et al., 2018). The application of multiple statistical techniques contributes to the reliability and rigor of the study, facilitating a deeper understanding of the intricate relationship between immune cells and prostate cancer (Hemani et al., 2018a). All analyses were conducted with the “TwoSampleMR” package (v.0.5.7) in R (v.4.3.0).

## Results

The main results of the analysis of the association between 731 immune cell types and the risk of prostate cancer.

**FIGURE 3:**
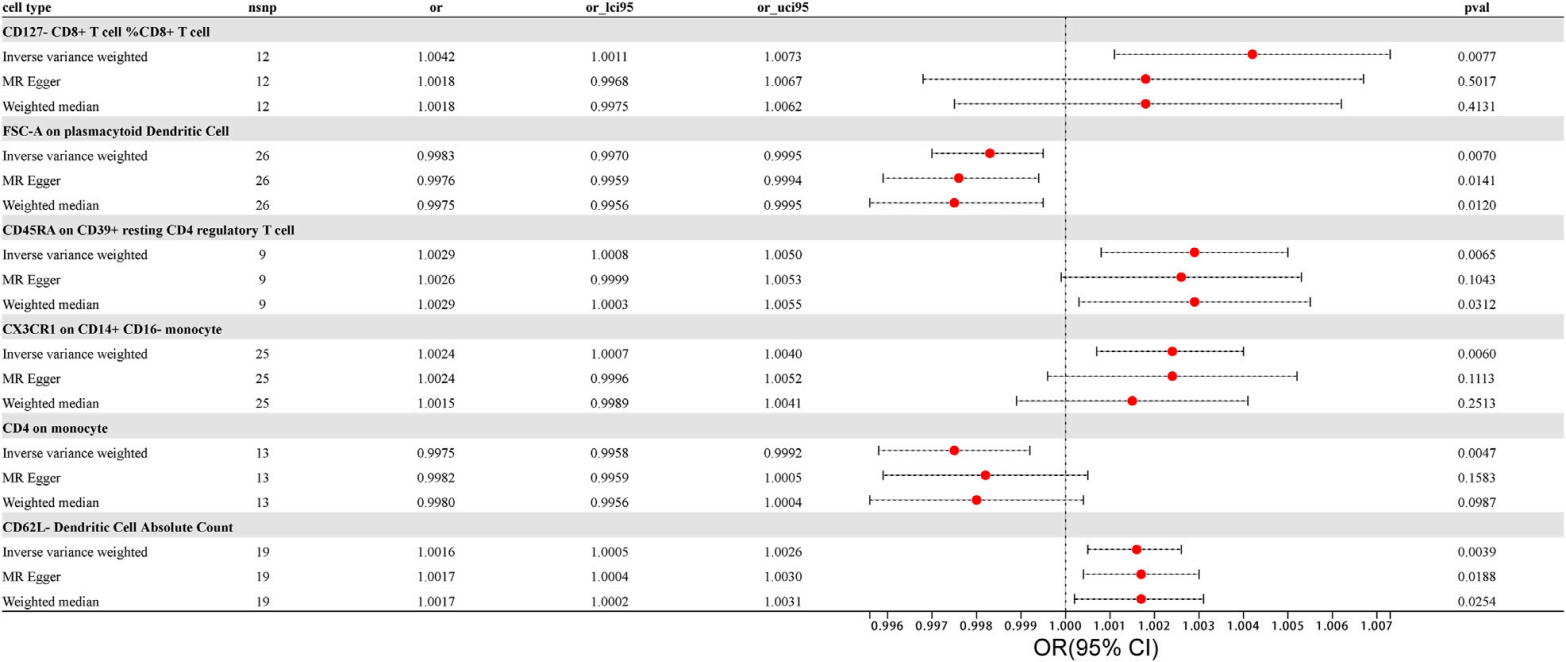
Forest Plot: Associations of Genetically Determined immune traits with prostate cancer risk.

F-statistics were calculated for all 731 immune cell types, ranging from 19.55 to 2381.77. The F-values for all results exceeded 10, surpassing the minimum threshold for weak instrument bias, indicating that they are all strong instrument variables. Detailed information on single nucleotide polymorphisms (SNPs) for each immune cell type is provided in Supplementary Table S1. The MR results for all features and their associations with prostate cancer are summarized in Supplementary Table S3, revealing six immune cell types with potential correlations detected using the IVW method, as shown in Figure 1. The IVs used for immune traits are presented in Supplementary Tables S1, S2. This MR analysis identified a causal relationship between six immune cell types and the risk of prostate cancer, as illustrated in Figure 3 and detailed in Supplementary Table S4. The study provides additional evidence to establish potential connections between specific types of immune cells and the risk of prostate cancer.

Using the IVW method, we found a clear association between T lymphocytes, monocytes-macrophages, dendritic cells, and the occurrence of prostate cancer. The IVW analysis revealed a positive correlation between CD127^-^ CD8^+^ T cell %CD8^+^ T cell and the risk of prostate cancer (OR = 1.0042, 95%CI:1.0011–1.0073, *p* = 0.0077). The MR-Egger and weighted median analysis methods did not reveal specific association. Both the MR-Egger intercept assessment (*p* = 0.2416) and MR-PRESSO global test (*p* = 0.3970) analysis did not indicate horizontal pleiotropy (Supplementary Tables S5, S6).

Similarly, CD45RA on CD39^+^ resting CD4 regulatory T cell, belonging to T cells, was confirmed to be positively correlated with prostate cancer risk through IVW analysis (OR = 1.0029, 95% CI: 1.0008–1.0050, *p* = 0.0065), with no significant association in weighted median and MR-Egger. No evidence of horizontal pleiotropy in the MR-Egger intercept assessment (*p* = 0.7244) and MR-PRESSO global test analysis (*p* = 0.8420) (Supplementary Tables S5, S6).

CX3CR1 on CD14^+^ CD16^−^ monocyte was identified through IVW analysis to be positively associated with the risk of prostate cancer (OR = 1.0024, 95%CI:1.0007–1.0040, *p* = 0.0060). This association was not significant in MR-Egger and weighted median analyses. Both the MR-Egger intercept assessment (*p* = 0.9813) and MR-PRESSO global test analysis (*p* = 0.8660) did not reveal horizontal pleiotropy (Supplementary Tables S5, S6).

For dendritic cells, CD62L^−^ Dendritic Cell Absolute Count was confirmed to be positively correlated with the risk of prostate cancer through IVW analysis (IVW: OR = 1.0016; 95%CI:1.0005–1.0026; *p* = 0.0039), with no significant association in MR-Egger and weighted median, and no evidence of horizontal pleiotropy in the MR-Egger intercept assessment (*p* = 0.7088) as well as MR-PRESSO global test analysis (*p* = 0.4650). The results, analyzed and tested using various methods while excluding outliers and heterogeneity, provided more accurate causal associations and offered new evidence in exploring which immune cells may promote the occurrence of prostate cancer (Supplementary Tables S5, S6).

In contrast to the cells positively correlated with the risk of prostate cancer mentioned above, we also identified immune cells negatively correlated with prostate cancer risk using the IVW method. In monocytes-macrophages, CD4 on monocyte was found to be negatively correlated with prostate cancer risk through IVW testing (OR = 0.9975, 95%CI:0.9958–0.9992, *p* = 0.0047). This correlation was not significant in MR-Egger and weighted median analyses, and the MR-Egger intercept assessment (*p* = 0.3962) as well as MR-PRESSO global test analysis (*p* = 0.7580) did not reveal horizontal pleiotropy. Similarly, in dendritic cells, FSC-A on plasmacytoid Dendritic Cell was identified through IVW testing to be negatively correlated with the risk of prostate cancer (OR = 0.9983, 95%CI:0.9970–0.9995, *p* = 0.0070). However, MR Egger and weighted median analyses did not find a significant association between these immune cells and the risk of prostate cancer, and intercept of MR-Egger analysis (*p* = 0.3206) as well as MR-PRESSO global test (*p* = 0.4020) analysis did not reveal horizontal pleiotropy in both cases (Supplementary Tables S5, S6).

**FIGURE 4:**
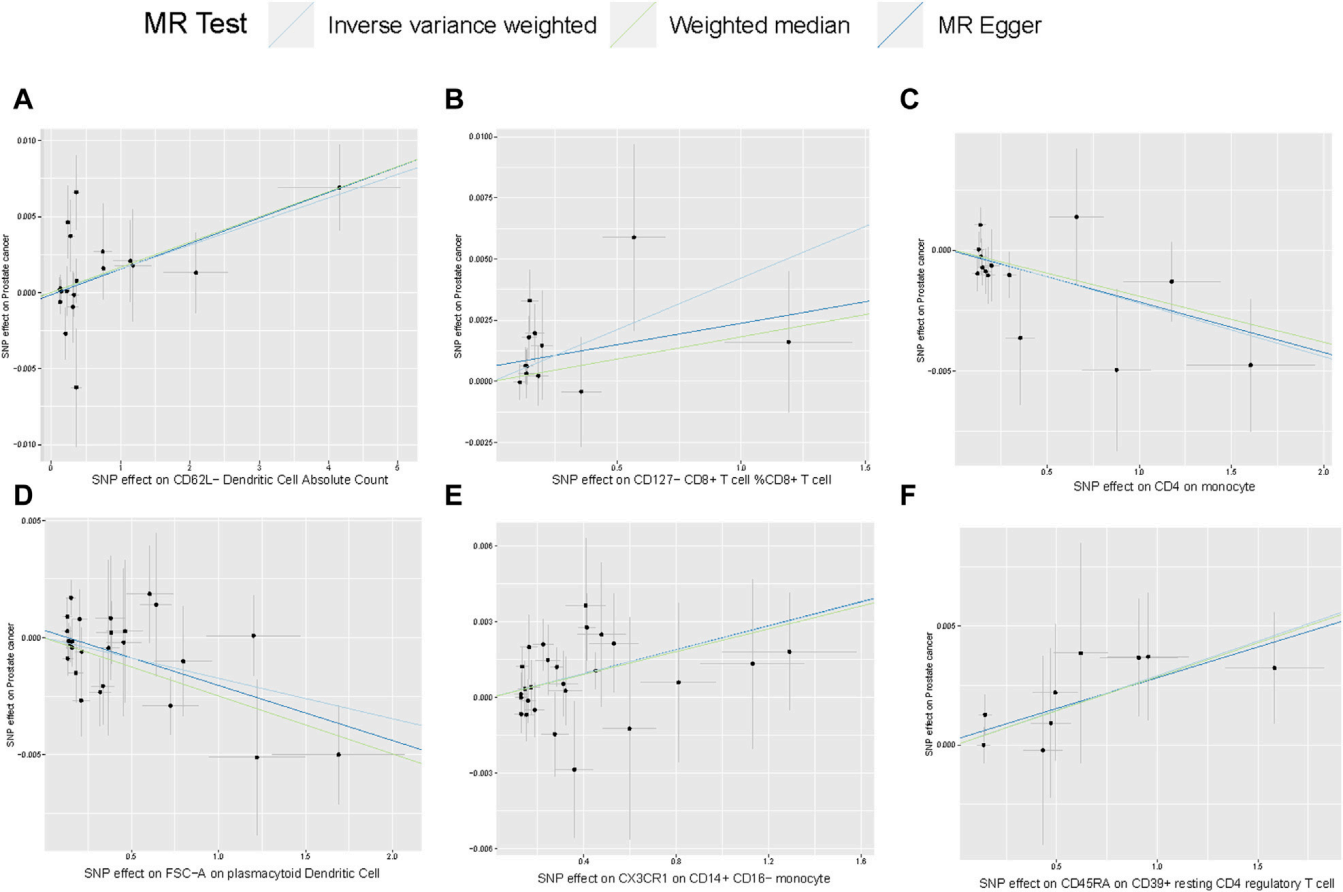
Scatterplot of genetic association between immune traits and prostate cancer. **(A)** Genetic association of CD62L− Dendritic Cell Absolute Count with prostate cancer Causal effect of Desulfovibrionales on CKD; **(B–F)** Potential causal effect of five other immune traits on prostate cancer.

Scatterplot of genetic association between immune traits and prostate cancer were shown in Figure 4. We found no significant heterogeneity among immune cells instrumental variables, which further indicates that immune cells play a complex and crucial role in the development of prostate cancer (Supplementary Table S7). Some cells promote the occurrence of prostate cancer, while others have the potential to inhibit the onset of prostate cancer. These findings provide new insights into the pathogenesis and treatment of prostate cancer.

## Discussion

In this MR analysis, we identified six types of immune cells related to the risk of prostate cancer, primarily including T cells, monocytes, and dendritic cells. These three categories exhibit different phenotypes and causal relationships with prostate cancer.

The study revealed a positive correlation between CD127^-^ CD8^+^ T cell %CD8^+^ T cell and the development of prostate cancer. CD127, also known as the IL-7 receptor alpha chain, is a component of the IL-7 receptor (Aloufi et al., 2021). IL-7 assumes a vital role in the progression of lymphocyte development (Lundstrom et al., 2012). In normal circumstances, immune cells, especially T cells, receive IL-7 signals through CD127, enhancing their survival and functionality to combat abnormal cells, including cancer cells. CD127^-^ cells, lacking the ability to receive IL-7 signals, result in immune cells becoming inactive or ineffective in the tumor microenvironment, leading to immune tolerance (Joshi et al., 2007). Therefore, the research findings indicating a positive correlation between CD127^-^ CD8^+^ T cell %CD8^+^ T cell and prostate cancer development are mechanistically reasonable. This provides a favorable basis for further exploring the exact role of CD127 in tumors.

In investigating the interplay between T cells and prostate cancer, we uncovered another intriguing result: CD45RA on CD39^+^ resting CD4 regulatory T cells is also positively correlated with prostate cancer development. CD45RA is typically expressed on unactivated, resting immune cells, especially unstimulated T cells (Hermiston et al., 2003). CD39, also known as NTPDase1, degrades extracellular ATP. Since ATP has pro-inflammatory effects outside the cells, CD39, by converting ATP to ADP and AMP, indirectly reduces extracellular ATP concentration, thus inhibiting inflammatory reactions (Timperi and Barnaba, 2021). Regulatory T cells (Tregs) suppress the activity of immune cells through various mechanisms.

In the tumor microenvironment, an excessive presence of Tregs may restrict the attack of other immune cells on tumor cells, promoting tumor escape (Ji et al., 2020). Therefore, CD45RA on CD39^+^ resting CD4 regulatory T cells, by degrading the pro-inflammatory factor ATP and inhibiting the anti-tumor effects of other immune cells, creates a more permissive immune environment for tumor cells. This allows tumor cells to evade immune system surveillance and attacks.

In the analysis of the interaction between immune cells and prostate cancer using MR analysis, a complex relationship was identified, such as a positive correlation between CX3CR1 on CD14^+^ CD16^−^ monocytes and the development of prostate cancer, and a negative correlation between CD4 on monocytes and prostate cancer development. CX3CR1 is a chemokine receptor that, by influencing monocyte chemotaxis and tumor angiogenesis (Pawelec et al., 2020), can alter the tumor immune microenvironment, thereby affecting tumor development and immune responses (Schmall et al., 2015). On the other hand, CD14 can interact with receptors such as TLR4, recognizing and binding to molecular patterns of bacteria (Marchesi et al., 2010). However, the role of CD14 in tumor development may be more complex. They can participate in anti-tumor immune responses (Pallett et al., 2023) and produce inhibitory cytokines that promote immune escape by tumors (Cheah et al., 2015). CD16 can bind to the Fc region of antibodies, forming complexes with antibodies. When these complexes bind to antigens on the surface of target cells, they activate natural killer cells, triggering ADCC (Bhatnagar et al., 2014). Based on the above, it is speculated that the possible mechanism by which CX3CR1 on CD14^+^ CD16^−^ monocytes promotes prostate cancer development is through the CX3CR1/CX3CL1 signaling pathway, promoting tumor angiogenesis, migration, and infiltration, while inhibiting ADCC, thereby weakening the body’s anti-tumor effects.

The expression of CD4 on monocytes is likely a marker of monocyte activation. Activated monocytes may participate in regulating immune responses, limiting tumor growth. However, as the tumor microenvironment changes (Zhen et al., 2014), the immune regulatory function of monocytes may be inhibited. The exact role of CD4 on monocytes in tumor development depends on the specific tumor type and individual differences among patients. Therefore, further experimental and clinical studies are needed. In summary, the MR analysis revealed complex interactions between monocytes and prostate cancer. This MR analysis is crucial for understanding the role of monocytes in cancer development and exploring new treatment methods.

Dendritic cells, a subset of antigen-presenting cells (APCs), play a crucial role in initiating and activating T cells, enhance the immune regulation of natural killer cells, and exhibit cytotoxic capabilities (Laginha et al., 2022). Presently, there have been encouraging outcomes observed in the use of immunotherapy utilizing dendritic cells for the management of prostate cancer (Jahnisch et al., 2010). This MR analysis provides the first confirmation that CD62L^−^ Dendritic Cell Absolute Count is positively correlated with the development of prostate cancer, while FSC-A on plasmacytoid Dendritic Cell shows a negative correlation. CD62L, also known as L-selectin, is a cell adhesion molecule that participates in leukocyte rolling, adhesion, and migration by binding to ligands on endothelial cells (Ivetic et al., 2019). Decreased expression of CD62L results in reduced chemotactic ability of dendritic cells, leading to a weakened anti-tumor inflammatory response. FSC-A is a crucial parameter used in flow cytometry to measure forward scatter signals and estimate cell size. Plasmacytoid dendritic cells are among the most potent regulators of antiviral immune responses in the body, producing large amounts of type I interferons (Mitchell et al., 2018), such as IFN-α. However, there is currently no experimental data supporting the negative correlation between FSC-A on plasmacytoid Dendritic Cell and prostate cancer. This finding provides new experimental avenues for exploring the relationship between dendritic cells and prostate cancer.

The intricate complexity lies in the interplay between prostate cancer and immune cells. These findings provide important insights into the roles of T cells, monocytes, and dendritic cells in the risk of prostate cancer, contributing to the advancement of immunotherapy for prostate cancer. However, there are certain limitations to consider. Firstly, the causal relationship between the identified six immune cells and prostate cancer was not strong. The causality links’ low power may result from the heterogeneity of the outcomes such as prostate cancer’s stage, severity, and duration. However, at this stage, there is still a lack of data on prostate cancer GWAS sequencing with specific clinical characteristics. Secondly, the population included in the Genome-Wide Association Study (GWAS) mainly comprises individuals of European ancestry. Genetic differences between populations may result in variations in the relationship between immune cells and prostate cancer, introducing a potential ethnic bias to the MR study results. Thirdly, the use of a low threshold value (*p* < 1.0 × 10^−5^) during the tool variable selection may lead to false positives or overlook important genetic variations related to immune cell features. Fourthly, the lack of independent cohort studies to validate the research findings. Fifthly, our research has only demonstrated partial correlation between immune cells and the development of prostate cancer, lacking experimental evidence to further explore and uncover the underlying mechanisms. In the future, we will conduct biological experiments to delve deeper into our findings and investigate potential mechanisms.

## Data Availability

The original contributions presented in the study are included in the article/Supplementary Material, further inquiries can be directed to the corresponding authors.
